# Effects of Cry1Ab Transgenic Maize on Lifecycle and Biomarker Responses of the Earthworm, *Eisenia Andrei*

**DOI:** 10.3390/s121217155

**Published:** 2012-12-12

**Authors:** Frances van der Merwe, Carlos Bezuidenhout, Johnnie van den Berg, Mark Maboeta

**Affiliations:** Unit for Environmental Sciences and Management, North-West University, Private Bag X6001, Potchefstroom 2520, South Africa; E-Mails: jfranvdm@gmail.com (F.M.); carlos.bezuidenhout@nwu.ac.za (C.B.); johnnie.vandenberg@nwu.ac.za (J.B.)

**Keywords:** earthworms, *Bacillus thuringiensis*, Bt maize, Neutral Red Retention Time, genotoxic, RAPDs

## Abstract

A 28-day study was conducted to determine the effects of the *Bacillus thuringiensis* Cry1Ab toxin on the earthworm *Eisenia andrei*. Previously, investigations have been limited to life-cycle level effects of this protein on earthworms, and mostly on *E. fetida*. In this study several endpoints were compared which included biomass changes, cocoon production, hatching success, a cellular metal-stress biomarker (Neutral Red Retention Time; NRRT) and potential genotoxic effects in terms of Randomly Amplified Polymorphic DNA sequences (RAPDs). NRRT results indicated no differences between treatments (*p* > 0.36), and NRRT remained the same for both treatments at different times during the experiment (*p* = 0.18). Likewise, no significant differences were found for cocoon production (*p* = 0.32) or hatching success (*p* = 0.29). Conversely, biomass data indicated a significant difference between the control treatment and the Bt treatment from the second week onwards (*p* < 0.001), with the Bt treatment losing significantly more weight than the isoline treatment. Possible confounding factors were identified that might have affected the differences in weight loss between groups. From the RAPD profiles no conclusive data were obtained that could link observed genetic variation to exposure of *E. andrei* to Cry1Ab proteins produced by Bt maize.

## 1. Introduction

Genetically modified (GM) Bt maize contains transgenes from the soil bacterium *Bacillus thuringiensis* that express insecticidal crystal (Cry) proteins. Until recently the most widely used event of Bt maize was event MON810 that expresses the Cry protein Cry1Ab. This protein is toxic for lepidopteron maize pests. In South Africa, the African maize stalk borer *Busseola fusca* (Lepidoptera: Noctuidae) is the primary target pest of Bt maize [1].

Expression of the Cry1Ab protein in maize plants takes place in the leaves, pollen, kernel and roots of the plant [2]. In contrast to the original proteins, truncated transgenic proteins are broken down much slower because they bind to surface-active particles, clays and organo-mineral complexes [3–6]. Breakdown is especially slow in soils that have a neutral pH in addition to high clay content, which facilitates the adsorption of the protein [4]. As a result of these properties, plant produced Cry proteins have the potential to accumulate in the soil [3–7]. The soil fate of the Bt protein is a key parameter governing exposure of non-target organisms in the environment [8]. The potential impacts of this protein on soil organisms further depend on persistence and its biological activity in soil [9]. Studies showed that Cry1Ab protein from Bt maize could persist in tropical soils for several weeks without losing insecticidal properties [10]. However, very short persistence periods have also been reported [8].

Of the main concerns regarding Bt crops is their potential effects on non-target organisms. Most studies on arthropods and soil microbial communities have failed to show significant negative effects [2,11–15]. This was also the case for earthworms (*Eisenia* spp.) where little to no difference has been observed with regards to growth and reproduction [5,8,13,16–18]. Studies in which another annelid, *Enchytraeus albidus* (Annelida: Enchytraeidae) was exposed to GM wheat with plant disease resistance did not show adverse effects on fitness of this species [19]. Results from a study in which *E. albidus* was exposed to maize leaves that expressed Cry1Ab protein provided inconclusive results [20].

In previous studies mortality or biomass reduction of earthworms were largely used as indicators of Cry protein toxicity but no studies have yet looked at toxicity on a cellular level. One such cellular marker is lysosomal membrane stability as measured by Neutral Red Retention Time (NRRT). This has already been illustrated utilising the earthworm *Eisenia* sp. as a bioindicator of toxins in soils [21–25]. The NRRT assay is based on the ability of viable coelomocytes, which occur in earthworm coelomic fluid, to incorporate and bind neutral red, a supravital dye, within their lysosomes. It gives an indication of the observation that membrane stability decreases in response to stress as membrane permeability increases. This assay has been shown to be a sensitive dose-responsive biomarker that indicates sublethal effects of pollutants on a cellular level before they become apparent on the earthworm life-cycle [22,24–28]. According to the assay, a decrease in NRRT indicates that the earthworm is experiencing stress.

Possible damage at the DNA level, caused by exposure of earthworms to Bt maize, has not been studied previously. Random amplified polymorphic DNA sequences is a PCR-based technique that has successfully been used in surveying genomic DNA for evidence of various types of DNA damage and mutational events in bacteria, plants, invertebrate and vertebrate animals [29–31].

The aims of this study were to determine both the changes in earthworm biomass and reproduction as well as the effects on earthworm NRRT when exposed to soil containing Bt maize leaf litter biomass that express transgenic Cry1Ab. The earthworm species utilised in this study was *E. Andrei*, in contrast to most other studies where metal pollution was investigated [23–25] which used *E. fetida*. Additional objectives were to determine the usefulness of lysosomal membrane destabilisation as measured by NRRT as a biomarker for stress related to Cry1Ab exposure as well as exploring the use of RAPDs to identify genotoxic effects of Cry1Ab.

## 2. Experimental Section

### 2.1. Rearing Substrates

Two maize isolines were used in the experiment *i.e.*, a transgenic maize variety (DKC 78-15B, transformation event MON810) expressing transgenic Cry1Ab proteins and its closest non-transgenic isoline (CRN3505) which does not express the protein. For both experiments only the leaves of maize plants grown in a greenhouse using a hydroponic system were used.

Commercial potting soil was used as rearing substrate for earthworms. This substrate contained peat, forest products and water-retentive agents, as well as slow-releasing fertilizer. Before adding fine maize leaves, soil was dried for 24 h at 60 °C and sieved using a 1,400 μm mesh. The leaves utilised in the trials were harvested at the 8 to 10 leaf stage, air-dried under greenhouse conditions and stored at room temperature. Maize leaves were shredded into smaller pieces before the start of the experiment using a food processor to a size of <0.5 cm^2^, after which it was stored at 4 °C until the start of the experiment. Confirmation that the Bt isoline contained Cry1Ab and that the non-Bt isoline lacked Cry1Ab was achieved by using QuickStix (EnviroLogix, Portland, ME, USA).

Adult *E. andrei* individuals with fully developed clitellums were used in the study. The soil mixture used consisted of 90% potting soil and 10% fine maize leaves (dry weight). For each treatment, 338 g of dried soil was mixed with 37.5 g fine, dried maize leaves. This volume of soil was then divided into three replicates of 124.5 g each. Soil was then wetted to field capacity (48% w/w).

Two treatment groups were used, one with soil containing Bt maize leaves (hereafter referred to as the Bt treatment) and the other containing non-Bt isoline maize leaves. The Bt treatment soil had a pH of 5.45 and the non-Bt treatment soil had a pH of 5.47. Three replicates of each treatment were used, each containing 10 adult *E. andrei* individuals. Rearing containers were kept in 24 h darkness at 25 °C and the first part of the experiment lasted for 28 days. During this time the biomass of each earthworm was measured once a week, as well as the NRRT of three earthworms from each replicate, after which earthworms were returned to the treatment soil. Earthworm biomass was measured by removing earthworms from the soil, washing them with distilled water and blotting them dry before weighing each worm separately.

At the end of the 28 day period the earthworms were removed from the soil. For the second part of the experiment the number of cocoons in each replicate were counted and then returned to the treatment soil. After a further 56 days the numbers of hatched earthworms were counted for each replicate. Throughout the experiment the moisture content for all replicates was kept constant.

### 2.2. ELISA Measurements of Maize Leaf Material and Soil

Soil samples were taken from both treatments before earthworms were added as well as at the end of the experiment after the hatchlings were counted. Samples that were taken at the start of the experiment were dried at 25 °C and kept at the same temperature until the ELISA was performed. The amount of Cry1Ab present in the four soil samples as well as the fine Bt maize leaves were determined using a Cry1Ab-specific ELISA kit (EnviroLogix).

The soil samples taken at the end of the experiment were dried for 24 h at 60 °C, and the dried soil and fine maize leaves were sieved using a no. 36 mesh (BSS, 422 micron). Centrifuge tubes (2 mL) were used and 0.1 g of each sample added to 1.5 mL extraction buffer (phosphate buffer saline (PBS), 0.55% Tween 20, pH 7.2) in the case of the maize sample, and 1 mL in the case of the soil samples. Tubes were vortexed and then centrifuged at 15,000 rpm for 2 min.

The maize sample was diluted by adding 100 μL of the sample extraction to 25 mL extraction buffer. Of the soil samples, all remained undiluted except for the sample taken from the Bt soil treatment at the start of the experiment, which was diluted by adding 100 μL of the sample extraction to 10 mL of extraction buffer. After dilution the samples were vortexed, 50 μL of conjugate (provided with kit) were added to the appropriate wells on the ELISA plate, after which 50 μL of the sample extract was added. Cry1Ab standards that were used contained a concentration series of 1, 2, 6, and 10 ng Cry1Ab/mL. These standards were used to draw a standard curve against which the samples would be compared. A blank control containing extraction buffer only was also included.

The plate was covered and mixed for 30 s on a plate shaker, after which it was incubated at room temperature while being mixed less vigorously for 1 h. The content of the wells were discarded and the wells were washed four times using wash buffer (PBS, 0.05% Tween 20, pH 7.2). All remaining liquid was removed from the wells by tapping the plate upside down on blotting paper. A 100 μL substrate solution (provided with kit) was added to each well, the plate was covered and incubated for 15 min at room temperature, after which a 100 μL stop solution (9% conc. HCl) was added to each well. The resulting colour changes were read on a spectrophotometer (450 nm) within 30 min, and sample results were plotted against the standard Cry1Ab concentration curve to determine sample Cry1Ab concentrations.

### 2.3. Lysosomal Membrane Stability Assays

Neutral red retention time (NRRT) was determined according to a method described by Weeks and Svendsen [32]. A neutral red stock solution was prepared by dissolving 20 mg of neutral red (toluene red, C_15_H_17_N_4_Cl) in 1 mL of dimethyl sulfoxide (DMSO, C_2_H_6_OS). Ten μL of the stock solution was added to 2.5 mL of physiological earthworm Ringer and mixed to give a working solution with a concentration of 80 μL·mL^−1^.

Coelomocytes to be examined for their NRRT were harvested by collecting coelomic fluid from clitellate earthworms used in the sublethal toxicity tests. This was done by extracting ±0.5 mL of temperature adjusted physiological earthworm Ringer (25 °C) into a syringe. An equal volume of coelomic fluid was extracted from earthworms. This was done by puncturing the body wall posterior to the clitellum with a syringe and extracting ±0.5 mL of coelomic fluid from the coelomic cavity. Afterwards, the worms were returned to their respective exposures after extraction of coelomic fluid.

Twenty μL of the suspension (coelomocytes plus Ringer) was placed on a microscope slide. The cells were left for 30 s to adhere to the slide surface. Twenty μL of the neutral red working solution was added to this suspension and a cover slip placed over it. Thereafter the coelomocytes were counted.

Each slide was examined continuously under a light microscope (×400 magnification) for two minutes with an interval of two minutes between the counting periods. During this period the number of observed live basophilic coelomocytes was recorded as well as those with fully stained cytosols (pinkish-red colour). The stained cytosols indicated the coelomocytes in which the dye had leaked from the lysosomes. The slides were kept in a humidity chamber to prevent desiccation during the two-minute intervals. Counting was continued until >50% of the counted coelomocytes had fully stained cytosols. This interval was recorded as the NRRT of the lysosomes in the specific cells.

### 2.4. RAPD Analysis

DNA was isolated using the DNeasy DNA isolation kit according to instructions of the manufacturer (Qiagen, Germantown, MD, USA). DNA quality and quantity was determined using a Nanodrop Spectophotometer ND-1000 v3.5.2 (NanoDrop Technologies, Inc., Wilmington, DE, USA). RAPD-PCR reaction mixes consisted of 2x PCR Master Mix (0.05 U/μL Taq DNA polymerase in reaction buffer, 4 mM of MgCl_2_, dNTP mix (dATP, dCTP, dGTP, dTTP each 0.4 mM; Fermentas Life Sciences, Waltham, MA, USA). Additionally, Supertherm Taq polymerase (1 U; JM Holdings, London, UK) and MgCl_2_ (1 mM final) were added to the 25 μL PCR mixture. Fifty nanograms of template DNA was used in each reaction. The primers used in this study are listed in Table 1. PCR conditions for each of the 40 cycles consisted of annealing at 37 °C for 30 s, primer extension at 72 °C for 1 min and denaturing at 95 °C for 30 s. An additional initial denaturation step of 95 °C for 300 s and a final extension of 72 °C for 300 s were included. A C1000TM Thermal Cycler (Bio-Rad, Hemel Hempstead Hertfordshire, UK) was used for PCRs.

Agarose 1.5% (w/v) was used for separating the RAPD fragments. Ten microliters of sample (DNA) and 3 μL 6× orange ladder loading dye (Fermentas Life Sciences) was mixed. Of this mixture 10 μL was loaded onto the gel. Each gel contained an O’GeneRuler 1 kb DNA molecular weight standard (Fermentas Life Sciences). Electrophoresis was conducted in a Wide Mini-Sub Cell GT Cell system (BioRad) for 150 min at 55 V. The electrophoresis buffer was 1 × TAE (40mM Tris, 1 mM EDTA and 20 mM glacial acetic acid, pH 8.0). A Gene Genius Bio Imaging System (Syngene, Synoptics, Cambridge, UK) was used to capture the image using GeneSnap (Version 6.00.22) software. Images were analyzed using GeneTools (Version 3.00.22) software (Syngene, Synoptics) to determine the positions and relative intensities of the bands in each lane.

For detection of potential genotoxic effects, RAPD profiles generated from control (unexposed) and treated (exposed) DNA were compared for missing bands or the appearance of new bands [30,31]. For this comparison worms were taken from a rearing colony and used as control treatment which was not exposed to maize in their diet.

### 2.5. Statistical Analysis

Changes in biomass and NRRT were analysed over time. One-way ANOVA and Kruskal-Wallis one-way ANOVA on ranks with Tukey’s post-hoc test was used, as well as Student’s t-test. Both changes over time within treatments and the differences between treatment groups over time were measured. The reproductive data, rates of biomass change as well as changes in RAPD profiles were analysed using Student’s t-tests. SigmaStat 3.1 (Systat Software, Inc., Richmond, CA, USA) was used for all analyses.

## 3. Results

### 3.1. Effects of Treatment Group and Exposure Time on Biomass

**Figure 1.**
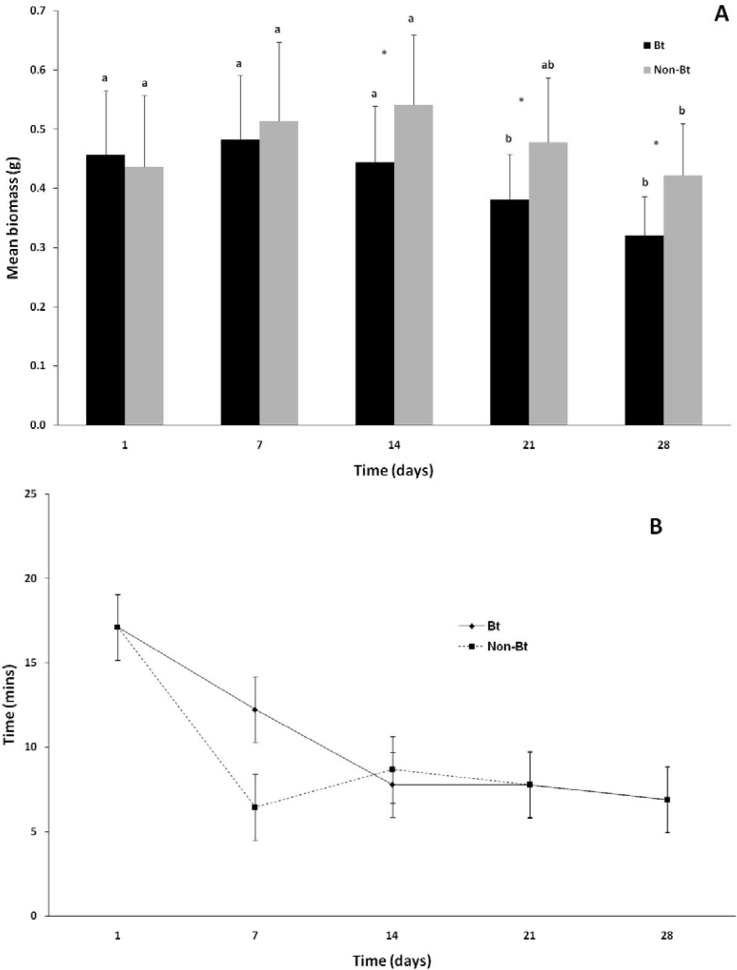
Changes in (**A**) earthworm biomass and (**B**) earthworm NRRT over the course of the experiment (means ± SD). Different letters between bars indicate a significant difference within a treatment between weeks. An asterisk (*) indicates significant differences between treatment groups.

The changes in earthworm biomass during the course of the experiment are indicated in Figure 1(A). There was no significant difference (*p* > 0.05) between the initial biomasses of the worms for the first 14 days of the experiment and that during week 1. Earthworms in both the non-Bt and the Bt treatment lost a significant (*p* < 0.001) amount of weight during the course of the experiment. In the first week of the experiment there was no significant difference in biomass between the Bt and non-Bt treatments (*p* > 0.50), but during the second week (day 14) and onwards a significant difference developed, with the Bt treatment losing more weight than the non-Bt treatment (*p* < 0.001). Worms in the non-Bt treatment showed a total decrease in body weight of 17.6% and those exposed to Bt a decrease of 33.3% (*p* < 0.05) over the duration of the experiment.

### 3.2. Effects of Treatment Group and Exposure Time on NRRT

Variance in the mean NRRT within treatments, especially in the Bt treatment, were high and no significant differences were observed either between treatments (*p* > 0.36), or within treatments over the course of the experiment (*p* = 0.18). The mean NRRT for the non-Bt treatment was 6.4 ± 4.2 min at the start of the experiment and 6.9 ± 3.3 min at the end of the experiment (Figure 1(B)). The mean NRRT for the Bt treatment at the first assessment was 12.2 ± 7.8 min and ranged between 7.5 and 6.9 during the course of the experiment.

### 3.3. Effects of Treatment Group on Reproduction

No differences (*p* = 0.29) in cocoon production between treatments nor the difference in numbers of earthworms that hatched between treatments were observed. The mean number of cocoons produced by worms exposed to non-Bt maize was 47.7 ± 13.7, and the mean number of earthworms that hatched from those cocoons was 51.3 ± 7.0. The mean number of cocoons produced by the Bt treatment was 56.7 ± 2.5, and the number of earthworms that hatched were 65.0 ± 18.2.

### 3.4. Effects of Treatment Group on RAPD Profiles Generated by 3 Primers

**Figure 2.**
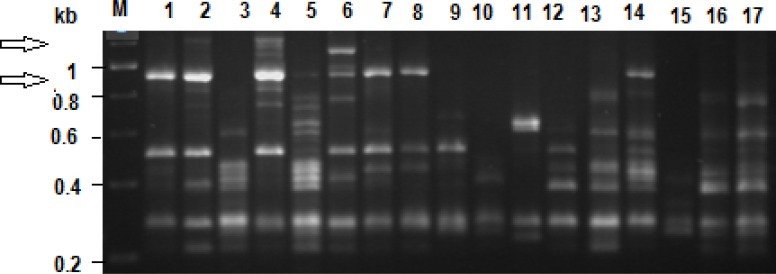
Ethidium bromide-stained agarose gel (1.5%, w/v) image showing the profiles obtained with one of the RAPD primers (OPA 18). Lane M represents the molecular weight marker (Fermentas, US). Lanes 1 to 5 are the profiles of individuals fed on Bt maize, 6 to 10 those that were fed non-Bt maize and 11 to 17 the control treatments. The arrows represent two loci (faint bands) that were associated with two individuals (lanes 2 and 4) fed on Bt maize.

Three primers were used and yielded a total of 341 scoreable bands (Table 1). Banding profiles, total number of bands as well as number of polymorphic bands was primer dependant and highly variable. However, in each of the profiles there was at least one band (locus) that was present in all representatives, indicating potential species markers for *E. andrei*. Overall, there were no significant differences between the Bt and the non-Bt fed individuals (t = 0.085) on the one hand, and the Bt fed individuals and out-group control individuals (t = 0.641) on the other. However, in the profile of two individuals that were exposed to Bt two faint loci were observed that were not seen in any of the those of the non-Bt fed maize and the control individuals (lanes 2 and 4 in Figure 2). These specific loci were not observed in the profiles other individuals that were fed on Bt maize.

### 3.5. ELISA Measurements of Maize Leaf Material and Soil

The Cry1Ab concentration in the fine Bt maize leaves incorporated into the Bt treatment was 17.13 μg/g. The Bt treatment had an initial Cry1Ab concentration of 142.5 ng/g, but at the end of the experiment no detectable Cry1Ab was left in the soil. No Cry1Ab was detected in the non-Bt or control treatment soil samples.

## 4. Discussion

In this experiment the soil pH was between 5 and 6, the ideal conditions for maximum adsorption of the protein to soil particles. The soil pH ideal for Cry protein adsorption is in the region of pH 5 to pH 7. As pH increases, adsorption of proteins to soil particles decreases and it is more easily broken down by microbes that utilize the toxins as a source of energy and carbon, while at a low pH the proteins tend to precipitate [3,4]. In a study that examined the rate of degradation in soil of Cry1Ab/c toxins originating from Bt maize biomass, Hopkins and Gregorich [33] found that all toxins degenerated within 14 days. It is therefore not surprising that by the end of this experiment no Cry1Ab could be detected, even though conditions were ideal for the adsorption of the toxin. Although a decline of Cry1Ab in the soil has been ascribed to earthworm activity [18], we cannot comment here on the effect that earthworms might have had on the decline of Cry1Ab in this study.

In the case of transgenic Cry proteins, accumulation can be especially detrimental for non-targets affected by the proteins since toxins can maintain their insecticidal activity throughout adsorption and desorption processes. Additionally, while the toxins are adsorbed onto soil particles they resist being broken down by microbes, being degraded by enzymes or being washed away by water [4]. Field studies that have investigated the amount of Cry1Ab present in the soil in fields where Bt maize (MON810) are grown have found that the toxin concentration ranged from 0.02 ng/g (wet weight) to 0.22 ng/g (dry weight) in the bulk soil and from 1.31 ng/g (dry weight) to 1.7 ng/g (wet weight) in the rhizosphere soil [2,34]. Compared to the starting concentration of 142 ng/g Cry1Ab in the soil of this experiment, it would seem that the concentration that the earthworms were exposed to is at least two orders of magnitude higher than the Cry1Ab concentration that would be found in the field. It must also be taken into account that our soil samples were not frozen until the ELISA was performed, but were kept at room temperature, possibly resulting in imperfect preservation of the toxin. Thus it is possible that the protein concentration was even higher originally than the measurement indicates.

Conflicting results have been reported on Bt protein persistence in soils. In a review of the environmental fate of Cry proteins done by Clark *et al.*[35] very short (<1 day) to medium persistence (21 days) were reported. Longer persistence was observed by Zwahlen *et al.*[6] who reported no further degradation of Cry1Ab protein in plant litter after 30 days in the soil. Persistence is however affected by soil physical-chemical properties. Studies showed that Cry1Ab protein from Bt maize could persist in tropical soils for several weeks without losing insecticidal properties [10]. Tapp and Stotzky [3] and Saxena and Stotzky [5] reported longer persistence with purified Bt toxin (>234 days) and root exudates of Bt maize (>180 days), respectively.

### 4.1. Organismal Responses

Jensen *et al.*[25] showed that following NRRT, growth (measured as change in biomass) and hatchability were the most sensitive endpoints to use when assessing the effect that a stressor has on earthworms.

Of five previous studies that reported on the biomass changes of earthworms exposed to Bt plant material [5,6,16,17,35], only one reported significant effects. In this study a decline in the weight of *Lumbricus terrestris* fed Bt maize compared to those fed non-Bt maize which only became apparent after 200 days of exposure to Bt maize [6]. No significant effects of Bt maize on *E. fetida* biomass have been reported [35]. This is of interest in the light of the species being used in this study being *E. andrei* which is closely related to *E. fetida*. The decrease of worm biomass observed in this experiment illustrates possible differences in species responses with regards to Bt maize since no other studies on *E. fetida* reported any effects.

If this result does indicate a non-target effect by Cry1Ab, this would mean that other transgenic maize events expressing Cry1Ab will probably also cause a non-target effect on *E. andrei*, since the MON810 transgenic event has resulted in Cry production that lies at the lower end of the spectrum of transgenic Cry expression [36].

It is important to acknowledge the presence of possible confounding factors that might have influenced the differential weight loss observed in the present study. Previous investigations have found that there was often a difference in the nutritional value of a transgenic Bt maize and its isoline, especially in the amount of lignin, sugar, protein and soluble carbohydrates in the plant’s tissues, with Bt maize often proving to have a better nutritional value that their isolines [7,11,35]. Some Bt isolines have been reported to have higher [7] or lower lignin content that their non-Bt isolines [11]. Increased lignin content in Bt maize leaves can cause a slower rate of breakdown of Bt maize compared to isoline maize, also possibly influencing the nutrients released by the leaves. It is highly likely that each transgenic event will cause different changes in gene expression in the transgenic plant compared to the isoline, and it is unknown which differences in nutrition exists for the maize isolines used in this study. If there are differences it is possible that it might have an influence on the observed changes in biomass.

The fact that both treatment groups lost a significant amount of weight during the course of the experiment can most likely be explained by the fact that maize leaves are not an optimal food source [35]. Comparing the rates of change in biomass between treatments showed that the rate of weight loss reached the exact same amount during the second week, and that no significant differences could be found between treatments.

Provided that cocoon production and hatchability are effective indicators of sublethal stress [25,28], the lack of significant differences in treatment groups possibly indicate that no genotoxic effects were caused by exposure to Cry1Ab protein produced by Bt maize. Further studies are however needed on this aspect.

### 4.2. Cellular and Genetic Responses

A review by Svendsen *et al.*[28] reported that when comparing the sensitivity of endpoints of toxicity in earthworms, NRRT was more sensitive than cocoon production, which in turn was more sensitive than the survival of earthworms. This would indicate that if adverse effects were caused in earthworms by Cry1Ab, the NRRT assay would provide the first indication of this.

Even though the NRRT of earthworms has been shown to be affected by a wide range of stressors, some stressors did not result in a sublethal decrease in membrane stability [28]. Examples of the effect of a wide range of stressors on NRRT are: organophosphates (*Apporectodea caliginosa*) [37], 2,4,6-trinitrotoluene (*Eisenia andrei*) [22], industrial soils (*L. Terrestris*) [26], copper oxychloride (*E. fetida*) [24], metals, organic compounds and polycyclic aromatic hydrocarbons [28], acetochlor (*E. fetida*) [27] and abamectin (*E. fetida*) [25]. Since this is the first study that have examined the effect that a Cry toxin has on the NRRT of earthworms, the absence of a response does not necessarily mean that earthworms are not experiencing any stress. This could merely mean that stress caused by Cry1Ab does not result in an effect on the NRRT of earthworms.

### 4.3. RAPDs Response

RAPDs have been used for genetic diversity studies and have previously also been used to demonstrate genotoxic effects on various organisms [29–31]. A study by Nadig *et al.*[29] for example, addressed exposure of sunfish to pollutants occurring in streams and demonstrated the effects of pollutants on genetic diversity of sunfish occurring in these waters. Lui *et al.*[30,31] also used RAPDs to demonstrate genotoxic effects of cadmium on barley. There is a lack of literature on of the use of RAPDs for possible Bt genotoxicity in earthworms. The present study explored the potential usefulness of RAPDs as a biomarker of the environmental effects of Bt maize on the genetic diversity of soil organism such as earthworms. The inconclusive results from our study could be due to a number of confluent factors including the population size of assessed worms, number of RAPD primers tested and exposure time.

## 5. Conclusions

Exposure to Cry1Ab-producing Bt maize only had an adverse effect on biomass change and none of the other biological parameters measured and this study did not provide any data that could link Cry1Ab exposure to genotoxic effects in one generation of *E. andrei*. The latter in the light of the confounding factors discussed, e.g., nutritional value of the maize. Even though there was no response in membrane stability in this study, future studies should investigate the effect on other cellular parameters, since negligible growth and reproductive effects have been found and continuing to do only short term studies will probably not yield much additional useful results. Future studies should investigate multi-generation effects on the sub-organismal and organismal level.

## Figures and Tables

**Table 1.** Details of RAPD primers used in determining genetic diversity among *Eisenia andrei* populations that were exposed to Bt and none-Bt maize. (A = adenine; C = cytosine; G = guanine; T = thymine; kb = kilobase).

**No.**	**Primer**	**Sequence 5′-3′**	**C+G (%)**	**Total no. of scored bands**	**Unique bands in Bt treated**	**Bands size range (kb)**
1	OPA 4	AAT CGG GCT G	60	128	0	0.35–0.65
2	OPA 10	GTG ATC GCA G	60	113	0	0.20–1.20
3	OPA 18	AGG TGA CCG T	60	100	2	0.25–1.20
		TOTAL	60	341	2	
